# Thermal and Mechanical Properties of Concrete Incorporating Silica Fume and Waste Rubber Powder

**DOI:** 10.3390/polym14224858

**Published:** 2022-11-11

**Authors:** Muhammad Tahir Lakhiar, Sih Ying Kong, Yu Bai, Susilawati Susilawati, Izni Zahidi, Suvash Chandra Paul, Mavinakere Eshwaraiah Raghunandan

**Affiliations:** 1 Discipline of Civil Engineering, School of Engineering, Monash University Malaysia, Bandar Sunway 47500, Selangor, Malaysia; 2 Department of Civil Engineering, Monash University, Clayton, VIC 3800, Australia; 3 Department of Civil Engineering, International University of Business Agriculture and Technology, Dhaka 1230, Bangladesh

**Keywords:** silica fume, waste rubber powder, mechanical properties, thermal properties

## Abstract

Using waste rubber tires for concrete production will reduce the demand for natural aggregate and help to reduce environmental pollution. The main challenge of using waste rubber tires in concrete is the deterioration of mechanical properties, due to poor bonding between rubber and cement matrix. This research aims to evaluate the mechanical and thermal properties of rubberised concrete produced by using different proportions of rubber powder and silica fume. Ordinary Portland cement was partially replaced with silica fume by amounts of 5%, 10%, 15% and 20%, while sand was replaced by 10%, 20% and 30% with waste rubber powder. Tests were carried out in order to determine workability, density, compressive strength, splitting tensile strength, elastic modulus, thermal properties, water absorption and shrinkage of rubberised concrete. The compressive strength and splitting tensile strength of concrete produced using waste rubber powder were reduced by 10–52% and 9–57%, respectively. However, the reduction in modulus of elasticity was 2–36%, less severe than compressive and splitting tensile strengths. An optimum silica fume content of 15% was observed based on the results of mechanical properties. The average shrinkage of concrete containing 15% silica fume increased from −0.051% to −0.085% at 28 days, as the content of waste rubber powder increased from 10% to 30%. While the thermal conductivity of rubberised concrete was reduced by 9–35% compared to the control sample. Linear equations were found to correlate the density, splitting tensile strength, modulus of elasticity and thermal conductivity of concrete with silica fume and waste rubber powder.

## 1. Introduction

Rubberised concrete produced by incorporating waste rubber tire particles could promote sustainable development by reducing the demand for natural aggregates. Rubberised concrete demonstrates lower density, higher impact resistance and toughness, and better sound absorption and heat insulation than normal concrete. The properties of rubberised concrete are affected by the parameters of rubber particles, namely rubber content, rubber particle size, and surface conditions of rubber particles. The mechanical properties of rubberised concrete are negatively affected as the content of rubber particles increases [1,2,3], attributed to the poor bonding between rubber particles and the cement matrix and the low stiffness of rubber.

Previous studies have demonstrated that the mechanical strength of rubberised concrete reduced as the size of rubber particles increased. The size of rubber particles can be classified into (i) chipped tire rubber particles size ranging between 13 and 76 mm is used as coarse aggregate, (ii) crumb tire rubber particles with sizes ranging between 0.075 and 4.75 mm to replace fine aggregate, and (iii) ground rubber particles size ranging between 0.15 and 19 mm. Some studies reported using rubber powder in concrete where the size of the rubber is less than 1 mm [4]. The rubber powder is angular in shape with a smooth surface texture [5]. The apparent density and specific gravity of rubber powder were reported as 400 kg/m^3^ [6] and 1.1 [5,7], respectively. Rubber powder is used to improve the properties of aerated concrete [8], polymer concrete [9,10], high strength concrete [11,12,13] and ultra-high performance concrete [14]. Other than rubber particles, some studies demonstrated that using sea sand could reduce the demand for river sand in concrete production [15,16].

Eldin and Senouci [17] reported that compressive strength was reduced more when coarse rubber chips replaced coarse aggregates than fine aggregates were replaced by crumb rubber at the same rubber content. Topcu [18] observed a decrease of about 50% in the cylinder and cube compressive strength in the concrete mixed with fine rubber particles (0–1 mm). For the concrete with coarser rubber particles (1–4 mm), the cylinder and cube compressive strengths were reduced by nearly 60% and 80%. A similar trend was observed for concrete incorporated with rubber powder [19,20]. The finer rubber particles have a better void filling ability, and a relatively stronger bond between the finer rubber particles and the concrete matrix could be observed [20].

It is essential to note that tire rubber demonstrates both hydrophobic and non-polar characteristics, leading to poor bonding between the waste rubber and Ordinary Portland cement (OPC) in the concrete and reduced strength. Hence, the surface modification of waste rubber to enhance the rubber OPC bond is essential [21]. In order to improve the waste rubber OPC bond, tap water washing [22,23], surface treatment utilising silane coupling agent [22,24], NaOH solution and Ca(ClO)_2_ [25,26,27] have been investigated. The properties of rubberised concrete improved after the surface treatment of rubber chips and crumbs using silane coupling agents, NaOH solution, and Ca(ClO)_2_. Pre-coated rubber crumbs with OPC paste slightly improved the strength of rubberised concrete, whilst the ether solution reduced the strength of rubberised concrete [28]. The rubber particles coated with silica powder and water glass demonstrated better bonding strength with concrete [29].

Various surface treatment techniques have been investigated to improve the bonding between rubber powder and OPC matrix. Segre and Joekes [30] reported that the surface treatment of rubber powder using NaOH solution for 20 min improved the bonding to OPC paste based on the observation from SEM images. It was reported that NaOH was more effective for treating small sized waste tire powders than rubber chips [31,32]. Treating rubber powder with a silane coupling agent and carboxylated styrene butadiene rubber latex improved compressive strength and flexural strength by 4% and 13%, respectively, compared to the control concrete without rubber [33]. The partial oxidation treatment produced hydrophilic functional groups on rubber surfaces leading to the higher compressive strength of rubberised concrete compared to control samples without rubber powder [34,35]. A further study demonstrated that rubber powder oxidised with KMnO_4_ solution and then sulphonated with NaHSO_3_ achieved a 41.1% increase in the adhesion strength of the rubber and OPC paste [36]. The treatment of rubber surfaces with ethanol, methanol, and acetone [37] and ultraviolet radiation [38,39] improved the interfacial bonding of the OPC matrix and rubber powder.

Extensive research reported that replacing OPC with silica fume increases the packing density and densifies the interfacial transition zone between rubber particles (crumbs and chips), aggregates, and OPC matrix, leading to better bonding and, thus, enhancing the strengths of rubberised concrete [40,41,42]. The typical silica fume content adopted in previous studies was 10% as the OPC replacement [40,43,44], while other research adopted different silica fume contents [45,46,47]. Furthermore, the optimum content of silica fume depends on the OPC content and water-cement ratio [48]. Silica fume was reported to have adverse effects on the concrete slump and compressive strength [49]. The contradicting trends could be attributed to differences in the proportions of OPC, aggregates, rubber particles and surface conditions.

It is essential to conduct an experimental study to quantify the effects of different silica fume contents for rubberised concrete produced using different proportions of waste rubber powder. A comprehensive experimental study was carried out to analyse the thermal and mechanical properties of concrete containing different proportions of waste rubber powder and silica fume. The silica fume contents investigated were up to 20% at an increment of 5%. Three waste rubber powder contents considered were 10%, 20% and 30%. Waste rubber powder consisting of mesh 40 and mesh 80 was used in this research. The tests performed on rubberised concrete were workability, density, water absorption, compressive and splitting tensile strength, modulus of elasticity, shrinkage, and thermal properties.

### Research Significance

To overcome the challenges of depleting natural aggregate resources and environmental issues related to waste disposal, reprocessing automotive scrap tires into aggregates for concrete production is a feasible means to promote sustainable development. It is essential to note that the waste rubber tire contains natural rubber, polybutadiene rubber, and styrene butadiene rubber, which are hydrophobic and non-polar. This leads to poor bonding between waste rubber aggregates and OPC in the concrete thus affecting the mechanical strengths. Various surface treatment methods such as silane coupling agent [50] and NaOH solution [51] improved the bonding between waste rubber aggregates and OPC, which contributed to better mechanical strengths. However, chemical treatment not only increases the production cost but is also hazardous to humans and the environment [27]. Previous studies [25,43,44,52] focused on the workability and mechanical properties of concrete incorporating rubber chips and fibre aggregate as a partial substitute for coarse aggregate. The use of rubber powder may achieve better concrete performance than rubber chips, due to the increase in surface area to promote bonding with the OPC matrix. Very limited research has been conducted to investigate the effects of concrete with different combinations of waste rubber powder and silica fume content. Silica fume is a pozzolan and a by-product of silicon and ferrosilicon alloy production. The filling ability and the pozzolanic reaction of silica fume could effectively improve the mechanical properties of rubberised concrete. However, different optimum proportions of silica fume have been reported in previous studies [40,44,52] and [45,46,47]. Thus, it is imperative to conduct a comprehensive experimental study to evaluate the mechanical and thermal properties of rubberised concrete produced using different proportions of rubber powder and silica fume. This study aims to provide insight into the effects of different rubber powder and silica fume content on the mechanical properties of rubberised concrete.

## 2. Materials and Experimental Methods

### 2.1. Materials

**Figure 1:**
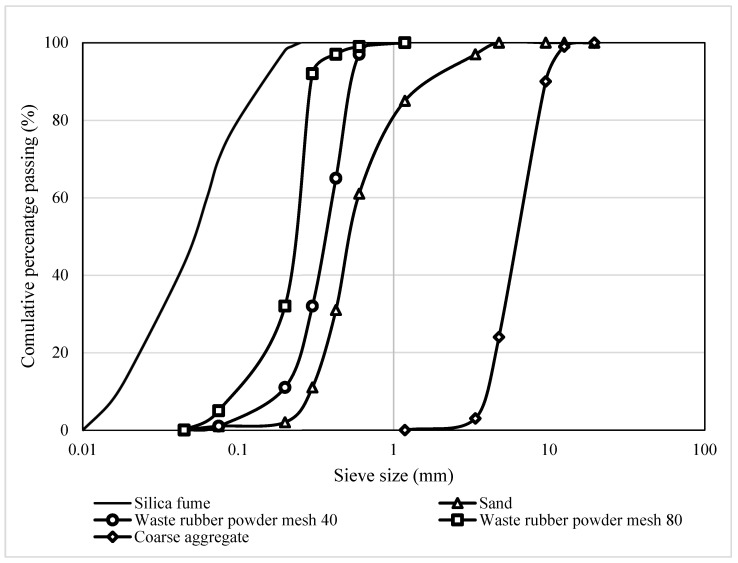
The particle size of sand, waste rubber powder and coarse aggregate distribution of aggregates.

**Figure 2:**
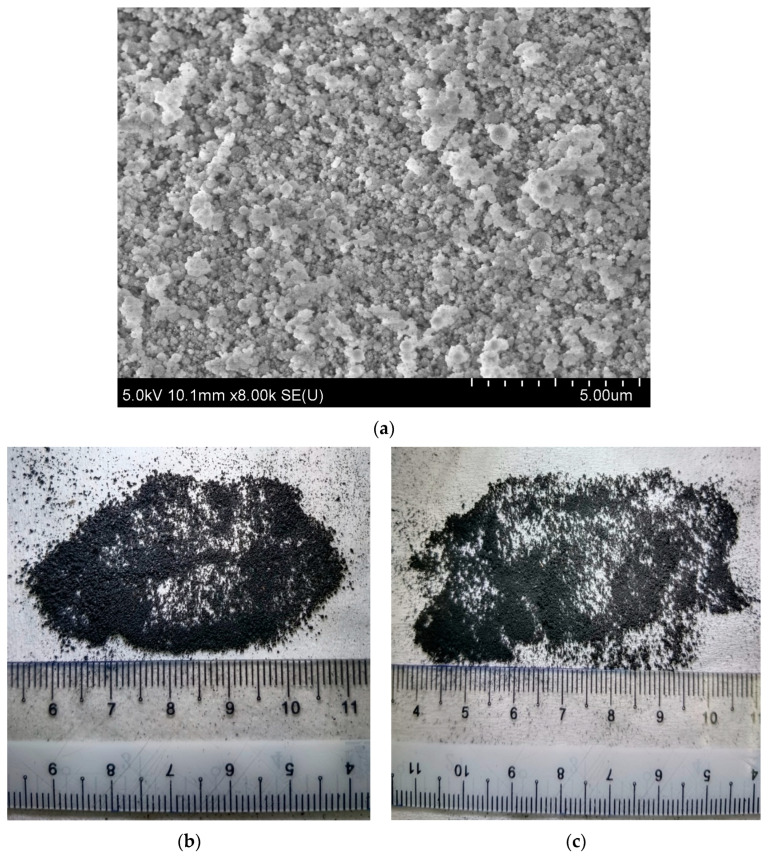
(**a**) Silica fume and waste rubber powder (**b**) mesh 40 and (**c**) mesh 80.

Type I, OPC with a specific gravity of 3.14 g/cm^3^ was used in this research. A local seller in Malaysia delivered the silica fume. The specific surface area and specific gravity of silica fume were 3176 cm^2^/g and 2.32, respectively. The particle size distribution of silica fume was analysed using the Mastersizer laser diffraction equipment. The particle size distribution results of silica fume are presented in Figure 1. The median particle size (D50) of the silica fume was 116 µm. Figure 2a presents the agglomeration of silica fume particles where the particles were spherically shaped with a rough surface texture. The X-ray powder diffraction (XRD) test was performed to evaluate the nature of silica fume. The results showed that silica fume was 47.3% amorphous and 52.7% crystalline in nature. In addition, the chemical composition of silica fume was evaluated using the X-ray fluorescence (XRF) test. This testing was carried out through an XRF machine (Bruker AXS S4) as described in ASTM C114 [53], and the result is presented in Table 1. The XRF results showed that the silica dioxide content was about 96% which was essential for the pozzolanic reaction. It could be observed that natural river sand passing through a 4.75 mm sieve was used as fine aggregate. The water absorption and specific gravity of fine aggregate were 1.12% and 2.62, respectively. The coarse aggregate with a maximum size of 10 mm was used to prepare concrete mixtures. The specific gravity and water absorptions were 2.72 and 0.54%, respectively. The sieve analysis was conducted following ASTM D6913 [54] for aggregates and rubber powder and the results are presented in Figure 1. Two sizes of waste rubber powder, mesh 40 and 80 (Figure 2b,c), were collected from a local supplier and used as partial sand replacement. The water absorption and specific gravity of mesh 80 waste rubber powder were 2.83% and 0.97, respectively. The water absorption of mesh 40 waste rubber powder was 2.75%, and the specific gravity was 1.03.

### 2.2. Mix Proportions

To investigate the effects of waste rubber powder and silica fume content in the rubberised concrete, 13 concrete mixtures were prepared by incorporating different proportions of waste rubber powder and silica fume. Silica fume was used as an OPC replacement for up to 20% by the weight of cement at an increment of 5%. Waste rubber powder was used as a sand replacement, and the range investigated was from 0 to 30% (by volume of total sand at an increment of 10%). Waste rubber powder was prepared by combining mesh 40 and mesh 80 sizes at a volume ratio of 1:1. The British standard [55] was adopted to determine the mix proportion of the control mixture with a targeted compressive strength of 30 MPa at 28 days. The mix proportions for 13 concrete mixtures were summarised in Table 2. The water binder ratio (w/b) ratio was 0.5 for all the mixtures. The specimens were named according to the content of silica fume and waste rubber powder. For instance, the 5SF-10R specimen was prepared by using 5% silica fume (SF) to replace OPC and 10% waste rubber powder (R) to replace sand.

### 2.3. Experimental Methods

The waste rubber powder was washed thoroughly using tap water for 10 min in a container to remove dust and organic materials. The waste rubber powder was kept at room temperature for 24 h until the surface dried state was attained. The mixing process was started by mixing dry ingredients in the concrete mixer until a homogenous blend was achieved. After that, water was added gradually, and the mixing was stopped once good consistency was observed. The workability of rubberised concrete was measured immediately using the slump cone according to ASTM C143 [56]. Three samples were prepared for all the tests performed in this study. After 24 h of casting, the specimens were placed into a water tank for curing.

Cylinders with 100 mm diameter and 200 mm height were prepared to measure rubberised concrete density and water absorption according to ASTM C642 [57]. The water absorption test was conducted by immersing samples in tap water at room temperature. The mass of the samples was recorded before immersion. The mass of the samples was measured at 7 and 28 days of immersion using a digital scale after the removal of free water on the surface of samples.

**Figure 3:**
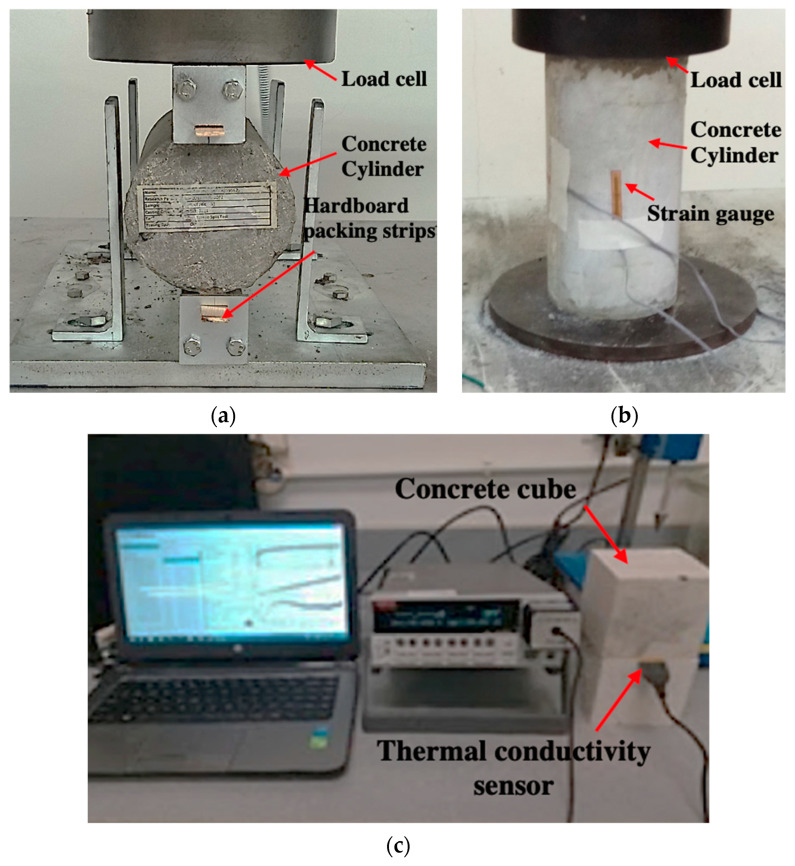
The testing of waste rubber powder concrete (**a**) splitting tensile strength, (**b**) modulus of elasticity and (**c**) thermal conductivity.

Concrete cubes of 100 mm were prepared to determine the compressive strength at 7 and 28 days, according to ASTM C39 [58]. The splitting tensile test was conducted on cylinders with 200 mm height and 100 mm diameter (Figure 3a) according to ASTM C 496 [59]. The elastic modulus of rubberised concrete was measured using cylindrical samples of 100 mm diameter and 200 mm height. The top and bottom surfaces of samples were capped with high strength gypsum in order to ensure uniform load distribution in accordance with ASTM C1617 [60]. A strain gauge of 20 mm long was attached to the middle of the cylinder to measure its strain in the loading direction, as presented in Figure 3b. The elastic modulus was calculated based on ASTM C469 [61]. The load control setting was adopted for the universal testing machine with a loading rate of 0.2 MPa per second. The microstructural analysis was performed using VPSEM, where the samples were coated with gold coating before analysis [62,63].

The concrete prisms with dimensions of 40 mm × 40 mm × 160 mm were prepared to monitor the shrinkage at 7 and 28 days using a vertical digital comparator according to ASTM C157 [64]. The initial length of samples was measured after the demoulding of the concrete. Finally, concrete cubes of 100 mm were prepared to conduct the thermal properties test using a C-Thermal TC kit, and the test setup is shown in Figure 3c. The transient plane source sensor was placed between two identical concrete cube specimens. An electric current was applied to heat the sensor. The increase in temperature was observed by C-Therm software in regressing the thermal conductivity of concrete samples following ISO 22007 [65].

## 3. Results and Discussion

This section presents the experimental observations and discussions of the workability, density, water absorption, mechanical properties, shrinkage, and thermal properties of concrete comprising various proportions of waste rubber powder and silica fume.

### 3.1. Workability

**Figure 4:**
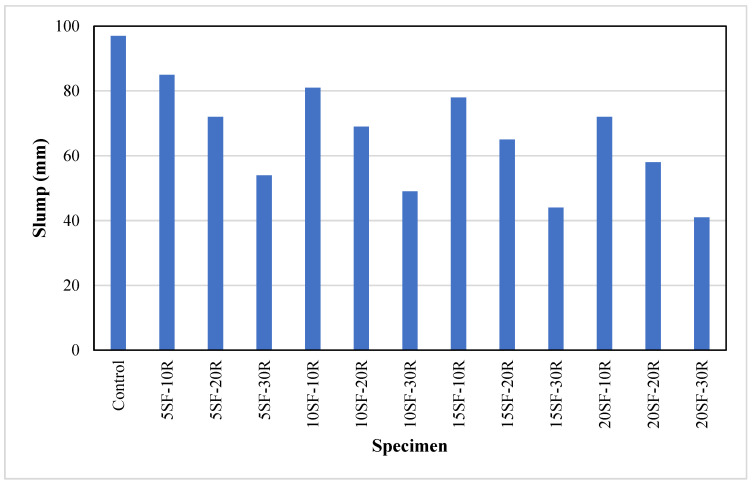
The workability of concrete with different proportions of waste rubber and silica fume.

Figure 4 shows the slump of concrete containing various proportions of waste rubber powder and silica fume. The control sample achieved a slump of 97 mm. The concrete slump reduced significantly as silica fume and waste rubber powder were used to replace OPC and sand, respectively. The results showed that the slump of concrete decreased from 85 mm (5SF-10R) to 54 mm (5SF-30R), indicating that replacing sand with waste rubber powder has a negative effect on the slump of concrete. These results demonstrated that the concrete containing 10% and 20% waste rubber powder achieved a slump in the range of 58 mm to 85 mm. While concrete with 30% waste rubber powder was categorised as low workable, as the slump observed was mostly below 50 mm. The decrease in the slump of concrete containing waste rubber powder was due to the rougher surface of rubber powder compared to sand which increased internal friction. Another reason reported by Ataria and Wang [66] that the slump of concrete with 20% rubber powder decreased by 11% compared to the control sample due to higher water absorption of rubber material than sand. Previous studies found a similar trend where the 20% to 80% slump decreased as the content of waste rubber powder increased from 5% to 20% in concrete [25,48].

Figure 4 shows that the slump decreased as the silica fume content increased from 5% to 20%. For instance, at 10% waste rubber powder content, the slump reduced from 85 mm (5SF-10R) to 72 mm (20SF-10R) as the silica fume increased from 5% to 20%. This reduction could be due to the higher water demand, as silica fume is finer than cement. These results agreed with the finding of the previous study [25] that the slump of concrete with 10% rubber powder decreased from 12.8% to 18.2% as the content of silica fume increased in concrete from 5% to 20%.

### 3.2. Density

**Figure 5:**
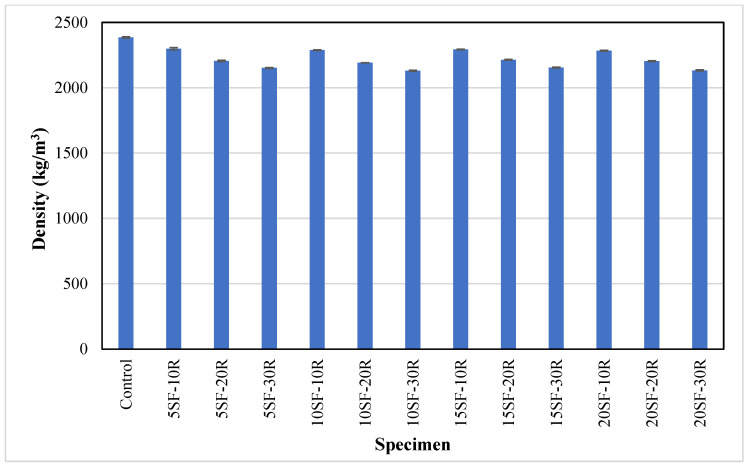
The density of concrete with different proportions of waste rubber and silica fume.

As presented in Figure 5, the average density of the control samples was 2385 kg/m^3^, and the replacement of sand with waste rubber powder reduced the density of concrete. As the waste rubber powder content increased in the concrete mix, the average density of concrete decreased in the range of 6% to 11%, depending on the rubber powder content. The lowest density of about 2100 kg/m^3^ was recorded by rubberised concrete with 30% rubber content. This reduction could be attributed to the lower specific gravity of waste rubber powder compared to the sand. Similarly, Wang et al. [67] also found a 26% and 28% reduction in concrete density with 10% and 15% rubber powder, respectively. Furthermore, the average concrete density marginally decreased as the silica fume content increased from 5% to 20%. This reduction in density may be due to the lower specific gravity of silica fume (2.32) to OPC (3.13). A similar trend was observed in the previous study [68], where the density of rubberised concrete was 16% lower at 10% silica fume compared to the control sample.

### 3.3. Water Absorption

**Figure 6:**
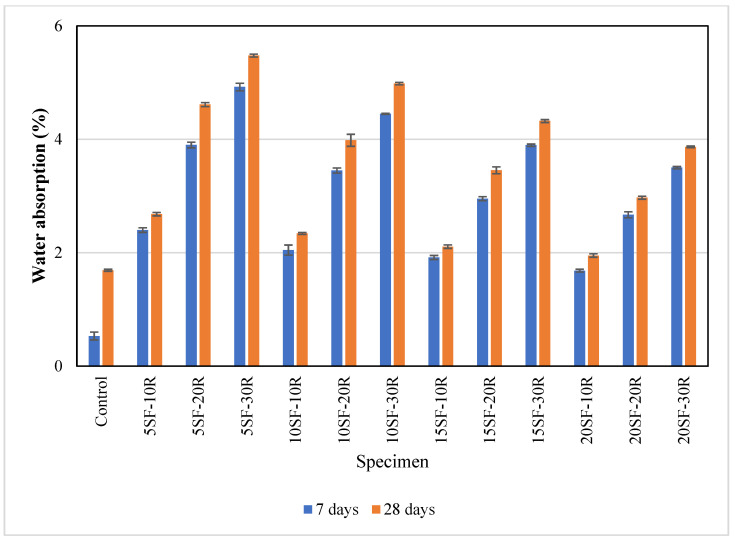
The water absorption of concrete with different proportions of waste rubber and silica fume.

Figure 6 shows the average water absorption results at 7 and 28 days. It could be observed that the average water absorption of the control specimens was 0.53% at 7 days and increased to 1.69% at 28 days. The average water absorption of the control specimens at 7 days to 28 days was low due to the strong bonding between the cement paste and aggregates.

**Figure 7:**
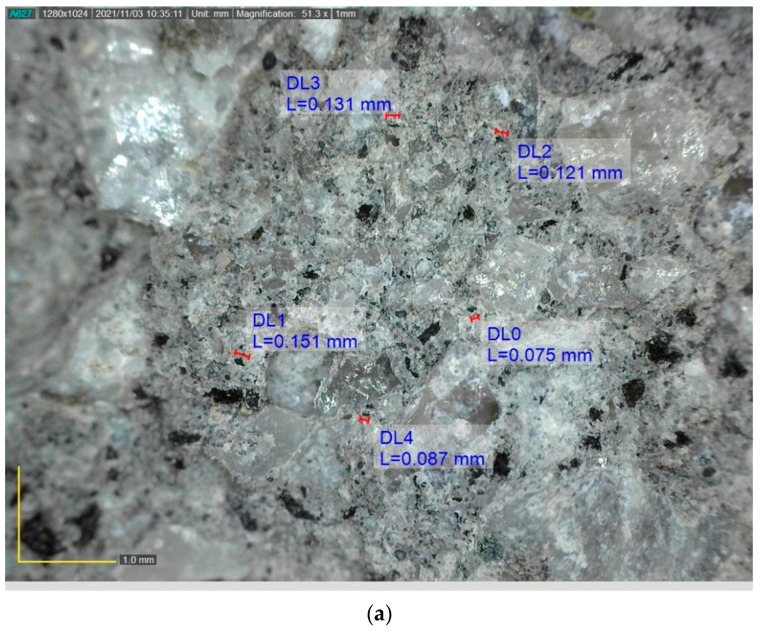

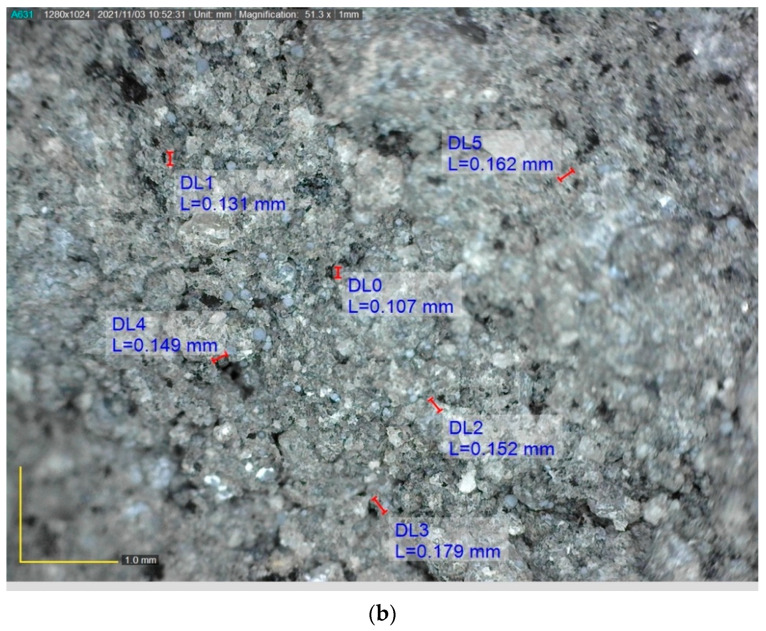
The microstructure of concrete with silica fume and waste rubber powder (**a**) 10SF-20R and (**b**) 10SF-30R.

The average water absorption of concrete with waste rubber powder was higher than the control specimens. The highest water absorption of 5.48% was observed in the 5SF-30R. The hydrophobic characteristic of waste rubber powder caused air to be trapped at the interfacial transition zone in concrete, leading to higher water absorption. Furthermore, workability reduced as the rubber content increased, which eventually led to the development of air voids. Figure 7 shows that higher air void content could be observed in 10SF-30R samples compared to 10SF-20R samples. Bisht and Rama [68] observed 26% water absorption increment as the percentage of rubber increased from 4% to 5% in the concrete mix. The rise in water absorption was due to the hydrophobic nature of rubber powder which resulted in the development of cracks and voids [69].

The replacement of OPC with silica fume reduced water absorption in concrete. For instance, the average water absorption of concrete (5SF-10R and 20SF-10R) decreased from 2.4% to 1.68% at 7 days, and 2.68% to 1.95% at 28 days as the content of silica fume increased from 5% to 20%. This was due to the pozzolanic reaction of silica fume, which reduced voids and pores by the supplementary hydration. As a result of the increased calcium silicate (CSH) gel generation, the concrete surface becomes more compact and has less porosity [40].

### 3.4. Compressive Strength

**Figure 8:**
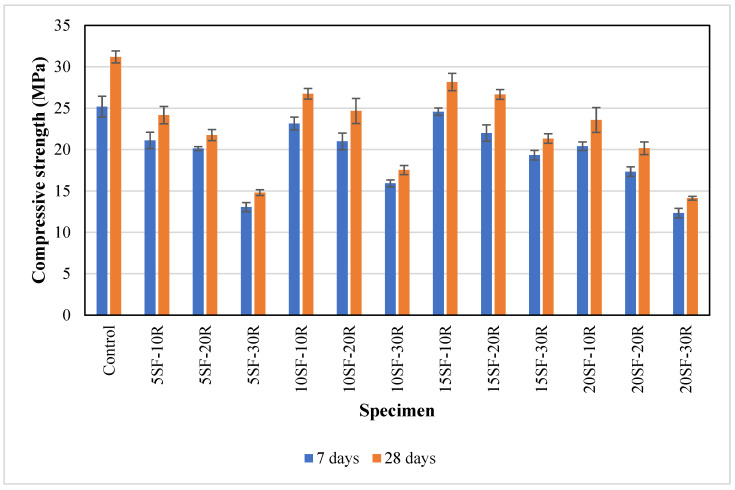
The compressive strength of concrete with different proportions of waste rubber and silica fume.

The average compressive strength of concrete containing silica fume and waste rubber powder at 7 and 28 days is presented in Figure 8. The average compressive strength of the control specimens was 25.2 MPa at 7 days, and it increased to 31.2 MPa at 28 days. It could be observed that the compressive strength was reduced as the waste rubber powder content increased up to 30%. The reduction trend could be observed irrespective of silica fume content. The compressive strength of about 15 MPa was observed for concrete samples (5SF-30R), which indicated a reduction of about 52% compared to the control samples. The reduction in compressive strength could be attributed to the low stiffness of rubber particles, which led to high internal tensile stresses and caused early failure in the concrete.

Furthermore, the hydrophobic characteristic of rubber particles caused weak bonding at the interfacial transition zone and reduced compressive strength. The decrease in the workability due to the increase in rubber content, as reported in Section 3.1, could lead to an increase in the air void content in the hardened concrete. The increase in the air voids could also lead to compressive strength reduction. A reduction in compressive strength of up to 50% was reported for concrete with a 15% replacement of aggregate volume using waste rubber chips [44]. The compressive strength was reduced by about 10% when 20% of rubber powder was used to replace sand for high strength concrete with a cement content of about 630 kg/m^3^ [20].

Overall, it could be observed that the compressive strength increased as the silica fume content increased for concrete with the same rubber content. The average compressive strength of concrete with 10% rubber powder increased by 10.6% and 16.6% when the silica fume content increased from 5% to 10% and 15%. Silica fume enhances the bonding between waste rubber powder and OPC by enhancing the interfacial transition zone, and leading to an increase in compressive strength [42,48,70]. Gupta et al. [42] found that the compressive strength of the rubberised concrete increased by 24.8% on 10% replacement of OPC by silica fume. However, it was worth noting that the compressive strength of rubberised concrete with 20% silica fume was lower than the concrete with 15% silica fume content at the same rubber content. Guneyisi et al. [48] reported that rubberised concrete with 20% silica fume demonstrated marginally higher compressive strength than rubberised concrete with 15% silica fume content. The different trends observed in this study could be attributed to the lower workability of rubberised concrete at 20% silica fume content which could lead to a higher air void content. The higher air void content in concrete may offset the strength increment due to the higher content of silica fume. Bisht and Rama [68] reported that the fall in mechanical properties was caused by voids generated due to the fineness of crumb rubber. Nevertheless, the compressive strength of 10SF-10R, 15SF-10R and 15SF-20R samples achieved a compressive strength of more than 25 MPa, which may be suitable for structural applications.

### 3.5. Splitting Tensile Strength

**Figure 9:**
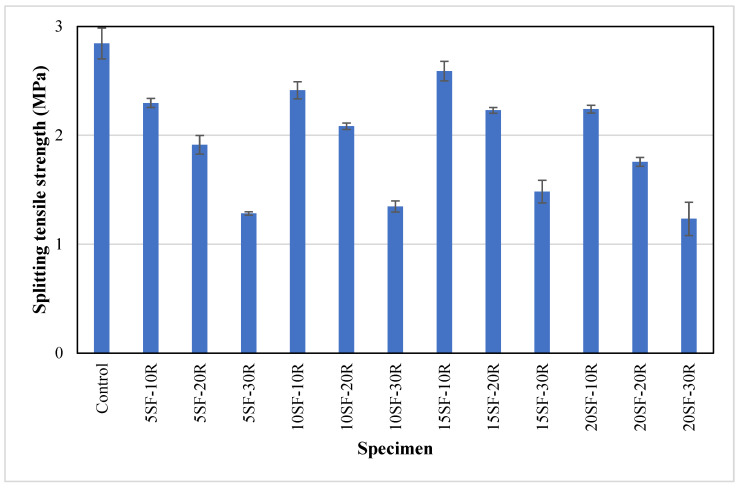
The splitting tensile strength of concrete with different proportions of waste rubber and silica fume.

Figure 9 presents the average splitting tensile strength of the concrete mixtures containing silica fume and waste rubber powder at 28 days. The average splitting tensile strength of the control specimens was 2.84 MPa. As expected, the splitting tensile strength of the concrete was reduced as the content of waste rubber powder in the concrete mix increased, irrespective of silica fume content. For instance, the average splitting tensile strength of the concrete containing 5% silica fume incorporated with 10%, 20% and 30% waste rubber powder was lower than the control sample by approximately 19%, 32% and 54%, respectively. The percentage of reduction in splitting tensile strength was similar to the percentage of reduction in compressive strength at the same replacement content of waste rubber powder. A similar trend observed in the previous study [20] that concrete with 20% rubber powder showed 8.3% reduction in splitting tensile strength compared to control sample. The possible reasons that led to the lower splitting tensile strength were the low stiffness of rubber particles, poor bonding at the interfacial transition zone and higher air content, as discussed in Section 3.4.

Figure 9 shows that replacing OPC with up to 15% silica fume enhanced the splitting tensile strength of concrete containing the waste rubber powder. At the waste rubber powder content of 10%, the average splitting tensile strength improved from 2.29 MPa (5SF10R) to 2.59 MPa (15SF10R) when the silica fume content increased from 5% to 15%. This showed an increment of about 13%. Guneyisi et al. [48] also reported the same trend where the splitting tensile strength of concrete increased by 12.1% to 42.6% as silica fume content increased from 5% to 15%. The increase in splitting tensile strength could be attributed to the higher fineness of silica fume particles and the development of CSH gel during the pozzolanic reaction, which filled concrete voids and improved bonding at the interfacial transition zone. The splitting tensile strength of rubberised concrete was reduced as the silica fume content increased from 15% to 20%, due to the possible increase of air void content, as discussed in Section 3.4.

### 3.6. Modulus of Elasticity

**Figure 10:**
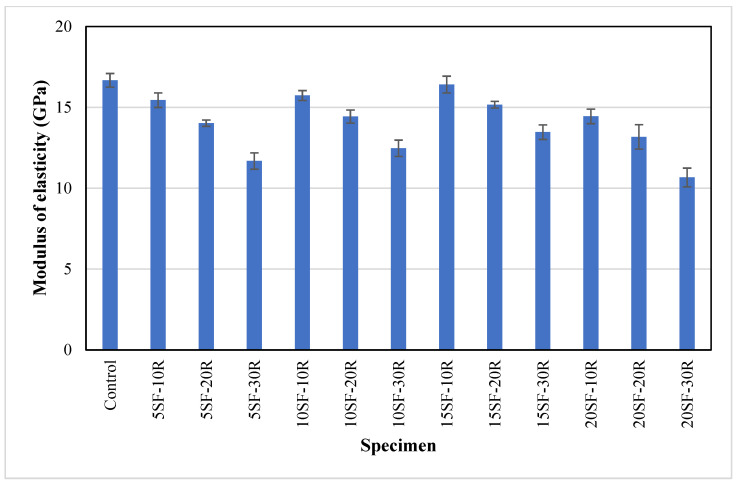
The modulus of elasticity of concrete with different proportions of waste rubber and silica fume.

The average modulus of elasticity for concrete containing waste rubber powder and silica fume is illustrated in Figure 10. The control specimens achieved the highest modulus of elasticity of 16.7 GPa. It could be seen that rubberised concrete showed a lower modulus of elasticity compared to the control specimens for all the combinations of silica fume and waste rubber powder content. The average modulus of elasticity of concrete decreased as the rubber content increased, and the reduction ranged from 1.6% to 36%. The reduction in modulus of elasticity could be attributed to the low stiffness of waste rubber powder, as the modulus of elasticity of concrete depends on the modulus of elasticity of aggregates and their volumetric proportions in the mix [42]. Similarly, Jalal et al. [44] observed a 32% reduction in the elastic modulus of concrete with 10% rubber chips compared to control samples. The replacement of OPC by silica fume up to 15% improved the modulus of elasticity of concrete with waste rubber powder, similar to the observations in the compressive strength and splitting tensile strength. The increment in the modulus of elasticity may be due to the development of CSH gel during the pozzolanic reaction, which enhanced the packing density of concrete [42]. It was noteworthy that 15SF-10R samples achieved identical modulus of elasticity compared to the control samples. These results also indicated that the use of waste rubber powder in concrete is more beneficial in improving the modulus of elasticity, rather than compressive strength and splitting tensile strength.

### 3.7. Shrinkage

**Figure 11:**
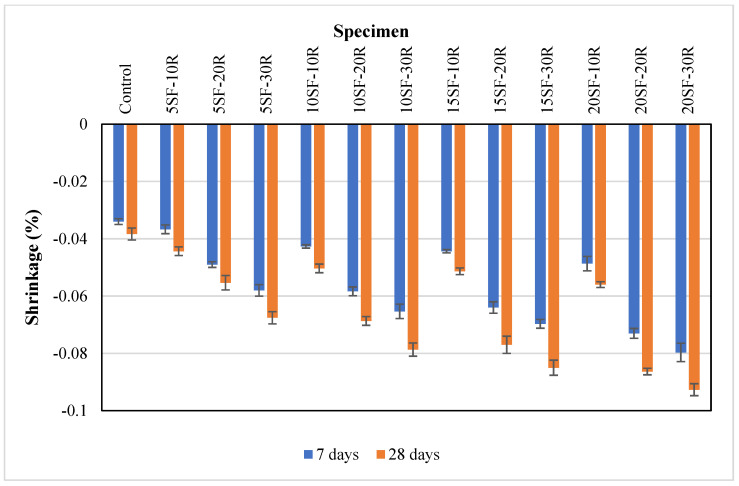
The shrinkage of concrete with different proportions of waste rubber and silica fume.

This experimental study measured the average shrinkage of concrete with silica fume and waste rubber powder at 7 and 28 days, as presented in Figure 11. The average shrinkage of the control samples was −0.034% at 7 days, and it increased slightly to −0.038% at 28 days. The average shrinkage of concrete containing 20% silica fume significantly increased from −0.048% to −0.079% at seven days and −0.056% to −0.092% at 28 days as the content of waste rubber powder increased from 10% to 30%. The increase in shrinkage could be due to the lower stiffness of rubber powder, which can undergo more significant deformation with minor internal stress compared to sand [27,71]. It was reported that the shrinkage of concrete increased from 35% to 95% as the amount of waste rubber powder increased from 5% to 20% in the concrete mix [72].

It could be observed that shrinkage increased as the content of silica fume increased. The average shrinkage at 28 days for concrete containing 10% waste rubber powder increased from −0.044% to −0.056%, as the silica fume content increased from 5% to 20%. It was reported that silica fume has a negative impact on the shrinkage of concrete as the refinement of pore size distribution led to a further rise in capillary tension and more contraction of the OPC paste [73]. It was found that the incorporation of silica fume significantly impacted the self-desiccation and shrinkage of OPC paste. Furthermore, the pozzolanic reaction of silica fume consumed the water content and led to shrinkage [74].

### 3.8. Thermal Properties

**Figure 12:**
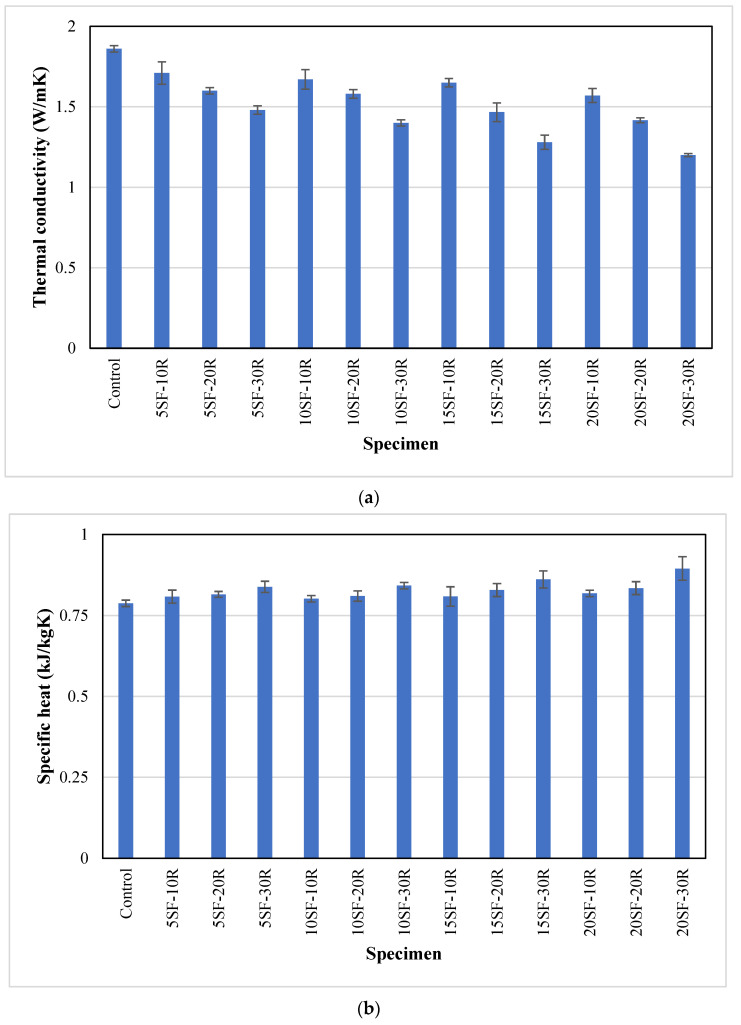
The thermal properties of concrete with different proportions of waste rubber and silica fume (**a**) thermal conductivity and (**b**) specific heat.

The thermal conductivity and specific heat of all concrete samples are presented in Figure 12a,b, respectively. It could be observed that the control samples showed the highest average thermal conductivity of 1.86 W/mK. The average thermal conductivity of concrete containing 5% silica fume decreased from 1.71 W/mK to 1.48 W/mK as the waste rubber powder content increased from 10% to 30%. A similar trend has been reported in the previous study [25], that the thermal conductivity of concrete with 8% rubber aggregates was 7.34% lower than the control mix. The lowest thermal conductivity of 1.2 W/mK was observed in 20SF-30R samples. The reduction in thermal conductivity of rubberised concrete could be attributed to the poor thermal conductivity of waste rubber powder compared to sand. Furthermore, fine rubber powder is more effective in heat insulation because of its high surface area that prevents more heat flux from transitioning [75]. The decrease in thermal conductivity of rubberised concrete when the percentage of rubber aggregate increased may be attributed to entrapped air and voids, as observed in Figure 7. The air content was caused by the porous texture of rubber aggregates [25]. The average thermal conductivity decreased within the range of 8% to 15% as the content of silica fume increased from 5% to 20%. Previous studies [76,77] reported that the thermal conductivity of concrete decreased by 2.5% to 10% as the content of silica fume increased from 10% to 30% in the concrete matrix. The reduction in thermal conductivity could be attributed to the finer particles of silica fume compared to OPC, which decreased the total porosity of concrete and improved their pore size distribution [78].

The average specific heat capacity of concrete containing silica fume and waste rubber powder was higher than the control sample. This could be attributed to the higher specific heat capacity of silica fume (750 J/kgK [79]) and waste rubber powder (1700 J/kgK [80]) compared to sand (710 J/kgK [81]) and OPC (720 J/kgK [81]). The average specific heat of the control samples was 0.79 kJ/kgK, while it was within the range of 0.80–0.89 kJ/kgK for concrete with silica fume and waste rubber powder. These results showed that an increase in the content of silica fume and waste rubber powder decreased the thermal conductivity while increasing the specific heat of concrete.

### 3.9. Morphology

**Figure 13:**
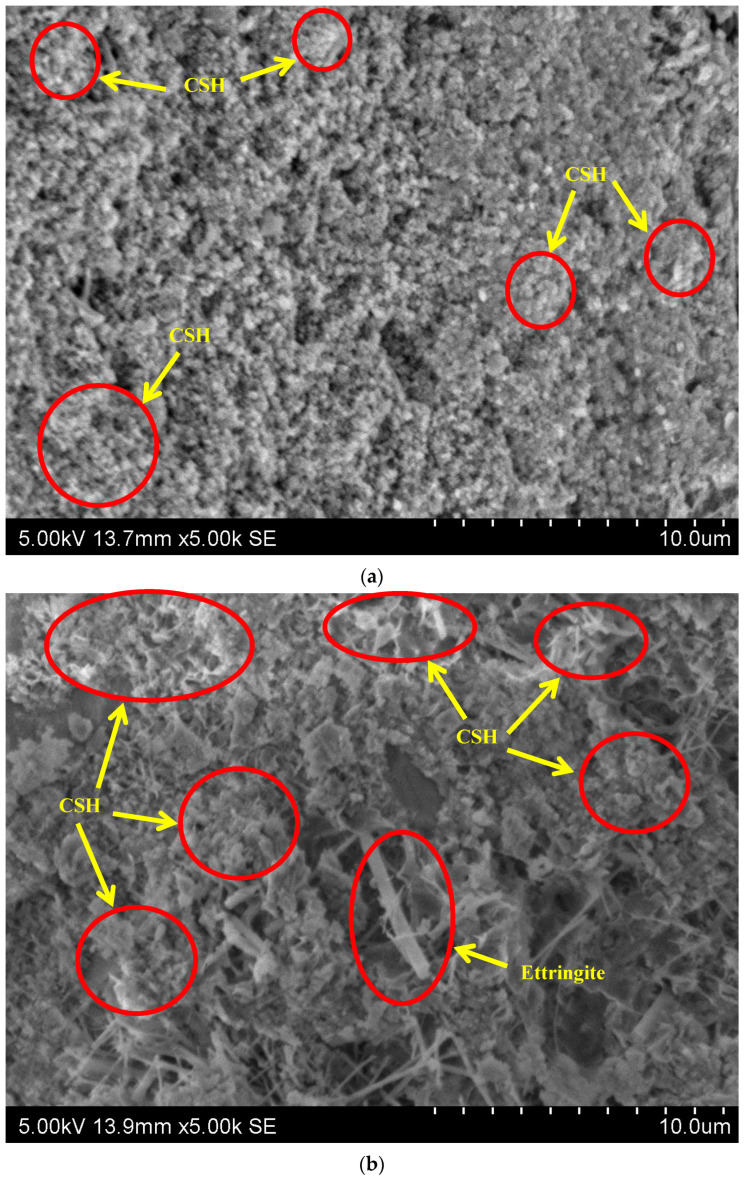
SEM images of concrete with different silica fume and waste rubber powder content (**a**) 5SF-20R and (**b**) 15SF-20R.

Scanning Electron Microscopy (SEM) was used to investigate the effects of silica fume on the microstructure of rubberised concrete. The microstructural images of concrete containing various proportions of silica fume are presented in Figure 13. The morphology of concrete containing 20% waste rubber powder at 5% silica fume content showed CSH gel (Figure 13a). Concrete with 20% waste rubber powder at 15% silica fume shows a higher density of CSH gel than 5% silica fume in concrete containing waste rubber powder, which leads to the compactness of the concrete matrix. Developing a high density of CSH improved the strength qualities by refining the microstructure. The ettringite was also observed in the concrete with 15% silica fume.

## 4. Statistical Analysis

The analysis of variance (ANOVA) is a statistical method for separating observed variance data into distinct components, so that additional tests may be performed. In this research, the ANOVA technique was used to evaluate the statistically significant parameters on the density, compressive strength, splitting tensile strength, elastic modulus and thermal conductivity of concrete containing the various percentages of silica fume and waste rubber powder. The ANOVA method was used to obtain F-value and *p*-value, in order to evaluate whether the effects of silica fume and waste rubber powder content were statistically significant or not significant. An alpha (α) level (error probability) of 0.005 was chosen for the ANOVA test. Furthermore, the *p*-value or probability value represents the probability for a specified statistical model that, when the null hypothesis is true, the statistical summary would be equal to or more extreme than the actual observed outcomes. While F-value is the ratio of change in sample means to change within samples. The larger the F-value, the more the difference between sample means relative to the difference within the samples. According to the previous study [44], the model may be statistically significant if the F-value is larger than F-critical, and the *p*-value must be lower than 0.05. Table 3 presents the ANOVA results for density, compressive strength, splitting tensile strength, elastic modulus and thermal conductivity of concrete with different proportions of waste rubber powder and silica fume. The ANOVA results showed that all the properties obtained a *p*-value less than 0.005 and an F-value greater than F-critical, which indicated that varying the proportions of silica fume up to 20% could affect the properties of concrete with rubber content up to 30%. In addition, as the waste rubber powder content increased in analysis, the *p*-value decreased significantly, and F-value increased. This shows that waste rubber powder content has significantly affected the density, compressive strength, splitting tensile strength, elastic modulus and thermal conductivity of concrete.

### Correlationships between Density, Thermal and Mechanical Properties of Waste Rubber Concrete

**Figure 14:**
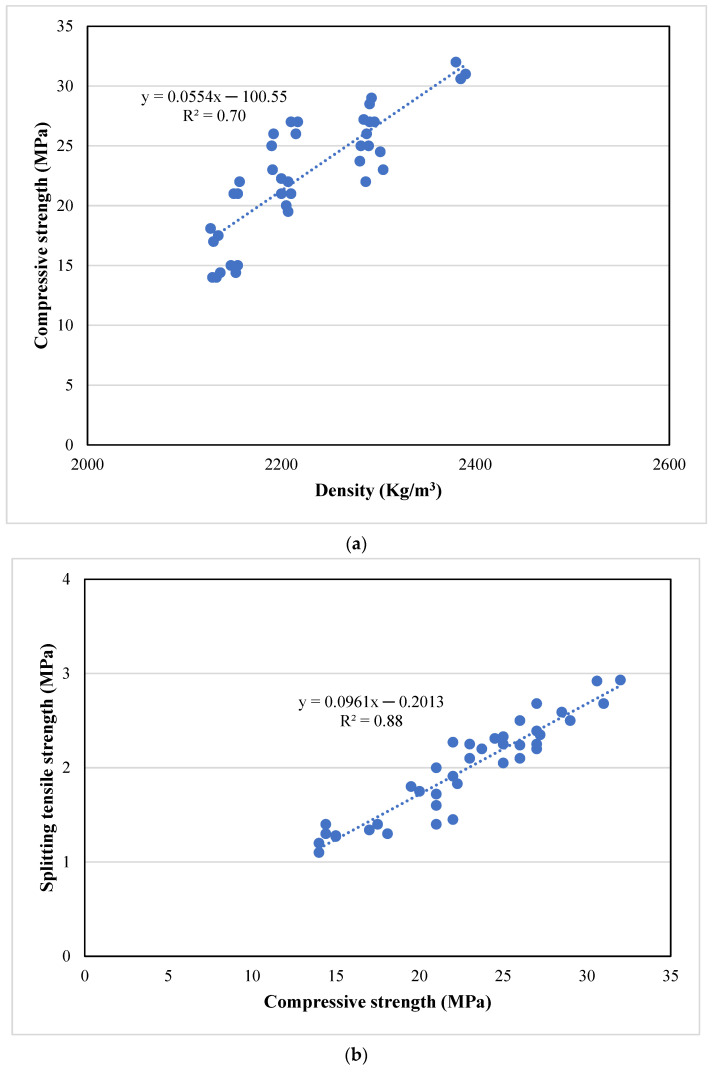

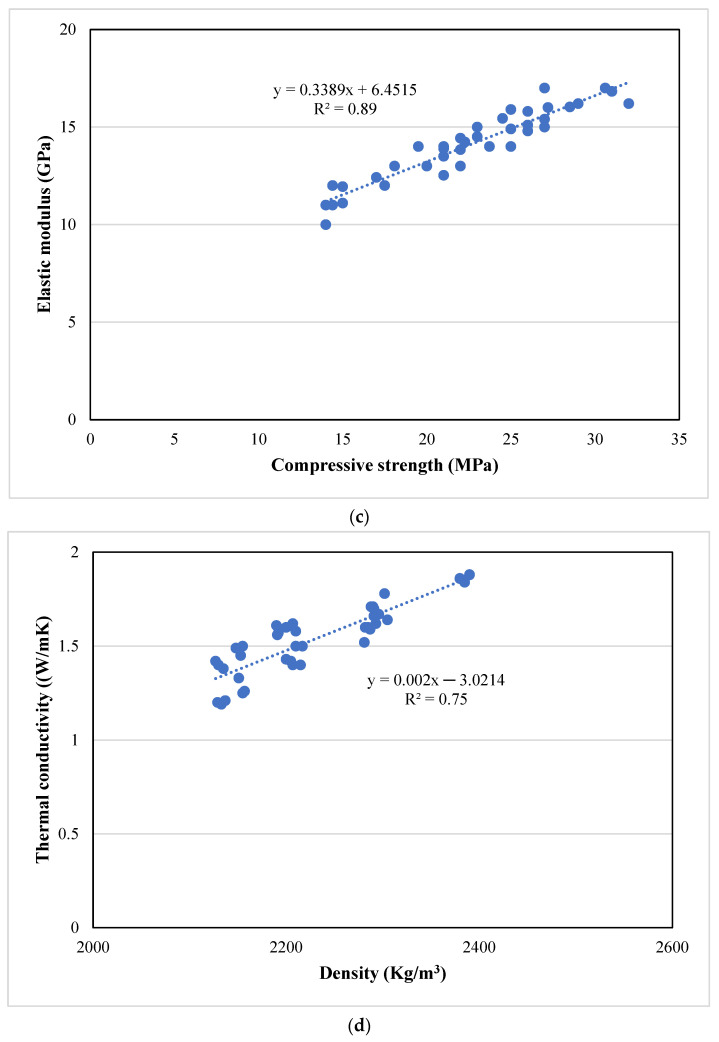

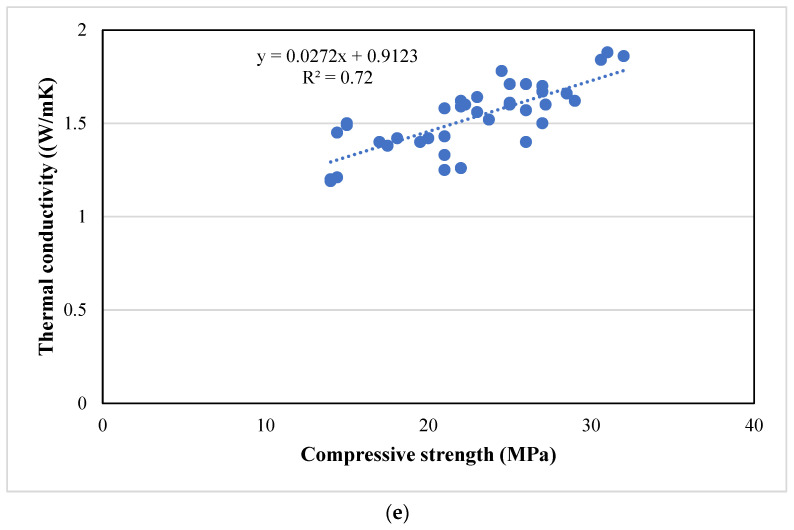
The linear relationships of rubberised concrete (**a**) compressive strength vs. density, (**b**) splitting tensile strength vs. compressive strength, (**c**) modulus of elasticity vs. compressive strength, (**d**) thermal conductivity vs. density, and (**e**) thermal conductivity vs. compressive strength images of concrete with different silica fume and waste rubber powder content.

The linear correlation technique was utilised to show the relationships between two quantitative variables. The linear model is one of the most widely used models in regression analysis because of its simplicity, practicality, and accuracy [82,83]. Previous studies [84,85,86,87] reported linear relationships between density and compressive strength for concrete with recycled rubber aggregate, different sizes of aggregates and lightweight aggregates. In addition, previous studies [85,87,88,89] found linear relationships between splitting tensile strength and compressive strength for concrete containing lightweight aggregate. While Eltayeb et al. [90], and Kockal and Niyazi [91], proposed linear relationships to correlate the modulus of elasticity and compressive strength for concrete containing rubber particles and lightweight aggregate. Furthermore, linear relationships were adopted to correlate thermal conductivity and density for concrete [52,92,93,94]. While Kazmi et al. [52] reported the linear relationship between thermal conductivity and compressive strength. Rashid et al. [83] and Ling [95] developed the linear relationships between density and compressive strength for concrete with rubber aggregate. Jalal et al. [44] showed that the linear relationship between compressive strength and elastic modulus for concrete containing silica fume and rubber chips could correlate well. It can be concluded from the literature that very limited studies have developed the relationships to correlate density with thermal properties and compressive strength for concrete containing different proportions of the silica fume and waste rubber powder content. Therefore, in this research, linear relationships were developed through the regression analysis for compressive strength vs. density, splitting tensile strength vs. compressive strength, modulus of elasticity vs. compressive strength, and thermal conductivity vs. density for concrete with silica fume and waste rubber powder at 28 days of curing, as shown in Figure 14.

The coefficient of determinant (R^2^) value obtained from the regression analysis shows the relevance between the regression curve and data points; the R^2^ value that was greater than or equal to 0.7 indicates a strong correlation between the test results [96]. Furthermore, the adjusted R-squared could further assess the adequacy of the equations. The adjusted R-squared signifies the difference between the rest of the experimental outcomes compared with the mean [97]. From Figure 14, it can be seen that the R^2^ values for various linear relationships range from 0.7 to 0.89. It shows that all linear relationships have a strong positive correlation between variables. A linear relationship was developed between the compressive strength and the density, as displayed in Figure 14a. R^2^ and adjusted R^2^ were 0.7 and 0.69, respectively, which confirmed the relationship’s applicability [98]. A previous study [84] developed the linear relationship between compressive strength and the density of pervious concrete. The results showed that the R^2^ value equals 0.62. In addition, Rashid et al. [83] found a similar linear relationship between compressive strength and density for concrete with rubber powder. The results defined the R^2^ value equals to 0.86. The developed equation is provided below:

f_cu_ = 0.0554*ρ* − 100.55; R^2^ = 0.70; Adjusted R^2^ = 0.69 (1)

where,

f_cu_ = Compressive strength in MPa
*ρ* = density in kg/m^3^

Figure 14b shows the linear relationship between compressive strength and splitting tensile strength of concrete with silica fume and waste rubber powder. A similar linear relationship and R^2^ value between compressive strength and splitting tensile strength for concrete are reported [84,89]. The linear equation is presented below:

f_ct_ = 0.0961f_cu_ − 0.2013; R^2^ = 0.88; Adjusted R^2^ = 0.87 (2)

where,

f_ct_ = Splitting tensile strength in MPa
f_cu_ = Compressive strength in MPa

Figure 14c exhibits the linear relationship between compressive strength and modulus of elasticity of concrete containing silica fume and waste rubber powder. The linear equation obtained from the present study is similar to Eltayeb et al. [90] for compressive strength and modulus of elasticity of concrete containing waste rubber powder. The linear equation obtained from this study is presented below:

E = 0.3389f_cu_ + 6.4515; R^2^ = 0.89; Adjusted R^2^ = 0.88 (3)

where,

E = modulus of elasticity in GPa
f_cu_ = Compressive strength in MPa

Figure 14d shows the linear relationship between the density and thermal conductivity of concrete containing silica fume and waste rubber powder. Kazmi et al. [52] also found a linear correlation between thermal conductivity and density and obtained a R^2^ value of 0.68. While Unal et al. [94] developed a similar linear relationship between the density and thermal conductivity of concrete with the R^2^ value of 0.82. The linear equation obtained from the current study is presented below:

(4) $$\lambda =0.002\rho -3.0214;\;R^{2}\;=\;0.75;\;\mathrm{Adjusted}\;R^{2}\;=\;0.74$$

where,

λ = Thermal conductivity in (W/mK)
*ρ* = density in kg/m^3^

Figure 14e displays the linear relationship between compressive strength and thermal conductivity of concrete containing silica fume and waste rubber powder. The previous study [52] also obtained a similar kind of linear correlation between thermal conductivity and compressive strength with the R^2^ value of 0.85. The linear equation found from this research is described below:

*λ* = 0.0272f_cu_ + 0.9123; R^2^ = 0.72; Adjusted R^2^ = 0.71 (5)

where,

λ = Thermal conductivity in (W/mK)
f_cu_ = Compressive strength in MPa

## 5. Discussion on the Optimum Content of Silica Fume

The compressive strength, splitting tensile strength and elastic modulus of rubberised concrete was improved using silica fume. The silica fume content has significant effects on the compressive strength and modulus of elasticity where the increment was about 15% when the silica fume content increased from 5% to 15% at a rubber content of 30%. The increment in the splitting tensile strength was less than 10% in this case. The optimum silica fume content of 15% was determined from this study as the mechanical properties of rubberised reduced as the silica fume content of 20% was adopted. Nagarajan and Shanmugasundaram [41] also reported that the optimum silica fume content of 15%. The addition of 15% silica fume in rubberised concrete showed 8% and 5% higher compressive and splitting tensile strength compared to samples with 20% silica fume at 5% rubber content. In addition, the compressive strength of rubberised concrete with 10% crumb rubber at 15% silica fume was almost 6% lower than the control sample [41]. The lower mechanical properties for concrete with 20% silica fume content could be attributed to the agglomeration of silica fume. Copetti et al. [45] observed that the concrete with 30% treated rubber at 15% silica fume showed almost 36% and 11% higher compressive strength and elastic modulus than concrete with 7.5% silica fume.

Different optimum silica fume content was reported in the previous studies. Gupta et al. [42] investigated the effects of silica fume content of up to 10%. It was reported that the concrete with 25% rubber fibres at 10% silica fume achieved higher compressive strength and elastic modulus than the concrete with 25% rubber fibres at 5% silica fume. The compressive strength of concrete with 25% rubber fibre increased by 33%, 27% and 25% for water binder ratios of 0.35, 0.45 and 0.55 when the silica fume content increased by 5% to 10%, respectively [42]. Guneyisi et al. [48] demonstrated that the compressive strength, splitting tensile strength and elastic modulus of concrete with 10% crumb rubber and 50% rubber chips were increased by up to 30%, 28% and 8% as the content of silica fume increased from 5% to 15% at 0.6 water to binder ratio. The difference between the mechanical properties of concrete with 10% crumb rubber and 50% rubber chips at 15% and 20% silica fume was insignificant (±5%). The improvement in mechanical properties was attributed to fine silica fume particles filling up concrete voids, which resulted in better adhesion between the rubber and cement matrix [48]. Moreover, Youssf et al. [99] reported the optimum silica fume content of 5%. The concrete with 20% rubber at 5% silica fume exhibited higher compressive strength (up to 7%) and splitting tensile strength (up to 9%) than the concrete with 10% and 15% silica fume. The higher silica fume content reduced mechanical properties of rubberised concrete may be due to the amount of SF that was added being more than the required amount for the filling mechanism and pozzolanic chemical action.

## 6. Conclusions

In this experimental investigation, rubberised concrete was developed by incorporating silica fume and rubber powder as partial replacements for OPC and sand, respectively. The rubber powder with Mesh sizes of 40 and 80 were used at a volume ratio of 1:1 to achieve better packing density. The workability, water absorption, shrinkage, and thermal and mechanical properties of rubberised concrete were investigated by varying the proportion of silica fume and rubber powder. Based on the experimental outcomes, the following conclusions could be drawn:

- The workability, mechanical properties and thermal conductivity of concrete decreased as the waste rubber powder content increased. Increasing rubber powder content led to higher water absorption and shrinkage. The partial replacement of OPC with silica fume by up to 15% improved the mechanical properties of rubberised concrete. Further increases in the silica fume content caused a reduction in mechanical properties. The ANOVA analyses confirmed that the silica fume and rubber powder content have significant effects on the properties of rubberised concrete.
- The mixture with a combination of 15% silica fume and 10% waste rubber powder was determined as the optimum proportion based on the mechanical performance. The reduction in compressive strength, splitting tensile strength and elastic modulus was less than 10%, and it achieved a compressive strength of more than 25 MPa. The thermal conductivity was 11% lower than the control sample.
- A minimum of 20% rubber powder was required as a sand replacement in order to achieve a thermal conductivity below 1.5 W/mK, which represented a 20% reduction in thermal conductivity compared to the control sample. With 30% rubber powder, a thermal conductivity as low as 1.2 W/mK could be achieved, but the compressive strength was less than 20 MPa.
- The proposed linear regression models could predict the relationships between splitting tensile strength, modulus of elasticity and thermal conductivity with compressive strength at an acceptable accuracy.

Even though this research presents comprehensive results on the mechanical and thermal properties of concrete with waste rubber powder and silica fume, further research on the long-term durability, flexural strength and impact resistance of concrete with waste rubber powder and silica fume should be conducted in order to provide a better understanding on these properties. In addition, the effects of combining silica fume with different surface treatment methods, such as NaOH and ethanol for waste rubber powder can be explored.

## Figures and Tables

**Table 1:** Chemical compositions of silica fume by XRF analysis (mass, %).

Chemical Composition	Silica Fume
Calcium Oxide (CaO)	0.26%
Silica dioxide (SiO_2_)	95.71%
Aluminium Oxide (Al_2_O_3_)	0.64%
Iron oxide (Fe_2_O_3_)	0.72%
Magnesium oxide (MgO)	0.41%
Potassium oxide (K_2_O)	0.24%
Sulphur oxide (SO_3_)	0.03%
Phosphorus pentoxide (P_2_O_5_)	0.05%
Loss of ignition (LOI)	1.94%

**Table 2:** The mix proportions of waste rubberised concrete.

Specimen	OPC (Kg)	SF (Kg)	Sand (Kg)	Waste Rubber Powder (Kg)		Coarse Aggregate (Kg)	Water (Kg)
				Mesh 40	Mesh 80		
Control	470	0	753	0	0	941	235
5SF-10R	470	23.5	677.7	14.9	13.3	941	235
5SF-20R	446.5	23.5	602.4	29.8	26.6	941	235
5SF-30R	446.5	23.5	527.1	44.7	39.9	941	235
10SF-10R	423	47	677.7	14.9	13.3	941	235
10SF-20R	423	47	602.4	29.8	26.6	941	235
10SF-30R	423	47	527.1	44.7	39.9	941	235
15SF-10R	399.5	70.5	677.7	14.9	13.3	941	235
15SF-20R	399.5	70.5	602.4	29.8	26.6	941	235
15SF-30R	399.5	70.5	527.1	44.7	39.9	941	235
20SF-10R	376	94	677.7	14.9	13.3	941	235
20SF-20R	376	94	602.4	29.8	26.6	941	235
20SF-30R	376	94	527.1	44.7	39.9	941	235

**Table 3:** The statistical analysis of thermal conductivity and mechanical properties of concrete with different proportions of waste rubber and silica fume.

Silica Fume Content	Waste Rubber Powder Content	*p*-Value	F-Value	F-Critical	Significance (*p*-Value < 0.05) and (F-Value > F-Critical)
Density					
5%, 10%, 15% and 20%	10%	7.2 × $10^{-10}$	238	3.5	Significant
	20%	1.7 × $10^{-13}$	1272.2		
	30%	1 × $10^{-14}$	2236.1		
Compressive strength					
5%, 10%, 15% and 20%	10%	2.4 × $10^{-5}$	26.9	3.5	Significant
	20%	3.5 × $10^{-7}$	66.8		
	30%	1.1 × $10^{-11}$	551.3		
Splitting tensile strength					
5%, 10%, 15% and 20%	10%	4 × $10^{-5}$	24.1	3.5	Significant
	20%	1 × $10^{-7}$	86.4		
	30%	1.9 × $10^{-8}$	121.8		
Modulus of elasticity					
5%, 10%, 15% and 20%	10%	7.1 × $10^{-4}$	12.3	3.5	Significant
	20%	2.7 × $10^{-5}$	26.4		
	30%	4 × $10^{-7}$	65.1		
Thermal conductivity					
5%, 10%, 15% and 20%	10%	3.3 × $10^{-4}$	14.8	3.5	Significant
	20%	9.7 × $10^{-8}$	87.5		
	30%	3.1 × $10^{-10}$	281.5		

## Data Availability

All the data are available within this manuscript.

## Nomenclature

Al_2_O_3_	Aluminium Oxide
ANOVA	Analysis of Variance
ASTM	American Society for Testing and Materials
Ca(ClO)_2_	Calcium hypochlorite
CaO	Calcium Oxide
CSH	Calcium silicate hydrate
Ca(ClO)_2_	Calcium hypochlorite
E	Modulus of elasticity
F_ct_	Splitting tensile strength
F_cu_	Compressive strength
Fe_2_O_3_	Iron oxide
ITZ	Interfacial transition zone
K_2_O	Potassium oxide
LOI	Loss of ignition
MgO	Magnesium oxide
NaOH	Sodium hydroxide
OPC	Ordinary Portland cement
P_2_O_5_	Phosphorus pentoxide
SEM	Scanning Electron Microscopy
SiO_2_	Silica dioxide
SO_3_	Sulphur oxide
XRF	X-ray fluorescence
*λ*	Thermal conductivity
*ρ*	Density
